# Isolation enhancement of a capacitively-fed MIMO antenna using a quasi-fractal parasitic element and defected ground structure

**DOI:** 10.1016/j.heliyon.2024.e39228

**Published:** 2024-10-12

**Authors:** Syed Naheel Raza Rizvi, Md Abu Sufian, Niamat Hussain, Yangbae Chun, Seong-Gyoon Park, Sunggoo Kim, Nam Kim

**Affiliations:** a Department of Information and Communication Engineering, Chungbuk National University, Cheongju, 28644, South Korea; b Department of Intelligent Mechatronics Engineering, Sejong University, Seoul, 05006, South Korea; c Department of Information and Communication Engineering, Kongju National University, Gongju, 314701, South Korea

**Keywords:** Vehicle-to-everything (V2X) communications, Vehicular internet of things, DSRC, ITS, MIMO antenna, Internet of things (IoT) antenna

## Abstract

Vehicular Internet of Things (IoT) is facilitated by efficient RF front ends with suppressed mutual coupling for enhanced spatial diversity and increased channel capacity. This paper presents a mutual coupling suppressed MIMO antenna with a hybrid decoupling technique for Vehicle-to-Everything (V2X) communications, enabling IoT in automotive systems. The single elements consist of a radiating patch with a cleaving circular slot to introduce a capacitive effect on the radiating structure. Afterwards, the single-unit design is further extrapolated to a 2 × 2 MIMO antenna. The mutual coupling is suppressed between antenna elements by introducing a quasi-fractal parasitic element and a defected ground structure (DGS). The MIMO antenna is designed to conform to the requirements posed by V2X systems in Dedicated Short-Range Communications (DSRC) and Intelligent transportation system (ITS) scenarios. The proposed MIMO antenna offers measured |S_11_| < −10 dB of 200 MHz, ranging from 5.77 GHz to 5.97 GHz, fully covering the spectrum guided by the IEEE 802.11p standard. A physical prototype is fabricated and placed on a car roof to assess the congruency between measured and simulated results. The MIMO antenna exhibits exceptional diversity properties, such as enhanced isolation (>28 dB) between its individual elements, a diversity gain (DG) close to the ideal value of 10 dB (9.99 dB), peak realized gain of 6.5 dBi, an ECC below 0.001, and a beam coverage area of 180° in azimuthal and elevation plane by dynamic port switching. Thus, the proposed MIMO antenna module is a considerable candidate for future V2X communication paradigms.

## 1 introduction

The dedicated vehicular communication systems are an emerging area of scholarly interest. Vehicular communications is a paramount area of research as it directly contributes to safe driving, autonomous vehicles, and the convenience of travel [[1], [2], [3], [4]]. By 2030, vehicular data and vehicular connectivity are projected to represent a value of 750 billion dollars, thereby representing significant financial and economic opportunities as well [5]. With the use of short-range communication signals, a vehicle could link with surrounding traffic and the environment as part of the Vehicle-to-Everything (V2X) communications paradigm [6]. The Federal Communications Commission (FCC) and 5G Automotive Association (5GAA) have concurred with the 5.9 GHz frequency spectrum for V2X communications, which is a direct spectrum allocation for automotive IoT. The spectrum from 5.77 GHz to 5.925 GHz encompasses all the frequency bands that are most often used internationally for V2X communications [7].

Multiple-input-multiple-output (MIMO) vehicle communication systems provide a wide range of techniques to increase signal reliability. This is because MIMO technology transmits signals over multiple channels of communication. The attenuation, delay, and phase shifts by the fading environment and the random processes on signal transmission are reduced when several communication channels are used. Additionally, the receiving end accurately executes the signal reception by combining the input from all the channels. Therefore, MIMO enhances the quality of vehicular communication as well as the channel capacity, reliability, and efficiency.

MIMO configurations allow several independent data transmission channels by utilizing multiple antennas. More information may be transmitted as an outcome of boosting the MIMO channel capacity. Enhancing MIMO channel capacity is a vital topic of research. In Ref. [8], a 10-element MIMO antenna is presented for improving the channel capacity for sub-6 GHz band communication. However, no solution for interference suppression is provided. Similarly, in Ref. [9], a MIMO antenna for automotive communications is presented without any solution for mutual coupling suppression. Effective signal transmission and reception are hampered by mutual coupling [10]. The electromagnetic interactions between the closely placed elements amplify the mutual coupling and result in reduced isolation [11], radiation pattern distortion [12], channel capacity reduction [13], and compromised reliability [14,15]. Thus, in a MIMO configuration with limited spacing between elements, mutual coupling reduction is an essential issue to address. For this purpose, many different methodologies have been used for mutual coupling reduction [[16], [17], [18], [19], [20], [21], [22], [23], [24], [25], [26]].

In [16], a three-element MIMO antenna is presented with self-decoupled probing for V2X communications. The minimum isolation offered by the design is more than 24 dB. However, the spacing between the antenna elements is not compact. In Ref. [17], field decorrelations are used in a MIMO antenna to offer an isolation of more than 16 dB. However, the isolation achieved by the design may not be suitable for V2X communications, which require high values of isolation [18]. utilizes a parasitic element to achieve high isolation at the cost of more interelement spacing [19,22]. offer self-decoupled patches for isolation enhancement. However, the spacing imposed by the design may restrict the use cases of the proposed MIMO antennas in IoT paradigms.

Recently, gap-coupled shortening strips have been utilized for generating decoupling waves in MIMO antennas. With the shorting strips, the resonant modes of the antenna can be controlled. In Ref. [21], gap-coupled shortening strips are used to generate decoupling waves for isolation enhancement. The design offers a minimum isolation of more than 13 dB. This technique can be applied to applications where decoupling wave generation can play a vital role in mutual coupling suppression.

Three-dimensional structures outside the main antenna geometry have been incorporated in MIMO antennas for mutual coupling suppression. In Ref. [23], an L-shaped metal wall is integrated with the main design geometry to enhance the isolation, and a reflector is incorporated at the rear side of the antenna to suppress mutual coupling in Ref. [24]. With improved complexity in design geometry, this integration can improve the isolation between individual elements. Furthermore, in Ref. [26], a microstrip resonator is etched between the antenna elements to enhance the surface current distribution and ameliorate the isolation between individual antenna elements.

Therefore, numerous methods can be used for mutual coupling suppression and isolation enhancement. These methods include self-decoupled probing, which is used in a three-element MIMO antenna for V2X communications. Field decorrelations are another technique utilized in MIMO antennas to achieve isolation. Parasitic elements are employed to achieve high isolation, although often at the cost of increased interelement spacing. Additionally, gap-coupled shortening strips are used to generate decoupling waves and control resonant modes. Three-dimensional structures, such as L-shaped metal walls and reflectors, are integrated with the main antenna design to enhance isolation. Furthermore, microstrip resonators are etched between antenna elements to improve surface current distribution and isolation.

In this paper, a MIMO antenna module is presented for V2X spectrum communications, specially encompassing the integration with IoT devices. The proposed antenna offers geometrically straightforward design architecture for mass-production, utilization of defected ground structure (DGS), and quasi-T shaped parasitic element for reduction of electromagnetic interactions between the antenna elements. This simultaneous use of DGS and parasitic elements results in mutual coupling suppression, a crucial requirement for safety-critical communication systems. The individual antennas are oriented to achieve 180 degrees of beam coverage. This targeted optimization for automotive environments, coupled with the achieved performance metrics—such as a peak realized gain of 6.5 dBi, a diversity gain close to the ideal value, wide beam coverage, and an envelope correlation coefficient (ECC) below 0.001—demonstrates an advancement over existing designs. The rest of the manuscript is as follows: Section 2 presents the design of the single element and MIMO antenna, whereas Section 3 presents the results of the proposed design. Section 4 is concerned with the comparison with previously published literature, followed by a conclusion and references at the end of the manuscript.

## 2 Design procedure of capacitively-fed MIMO antenna for vehicular iot

The proposed single element antenna operating at V2X spectrum is explained in this section. For the convenience of the reader, it is divided into sections as follows.

### 2.1 Single element antenna

**Fig. 1:**
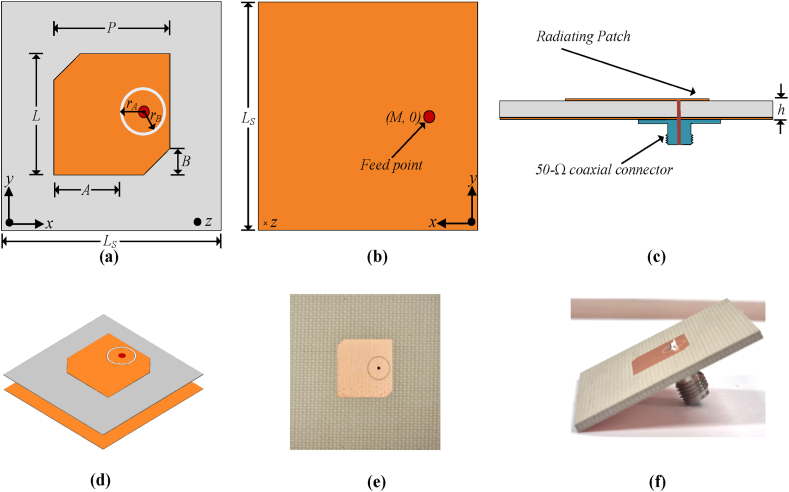
The geometry of the unit element of the proposed capacitively-fed MIMO antenna (a) front view (b) back view (c) side view (d) isometric view (e) fabricated prototype and (f) side view of fabricated prototype.

Fig. 1 represents the proposed geometry of the single unit of antenna. Initially, a square-shaped radiating patch is patterned on the substrate Rogers RO4350B ($\varepsilon_{r}$ = 3.66 and tanδ = 0.0037) with a height of 59.85 mils. A 50-Ω coaxial connector is used to feed the radiating patch. The antenna radiator is coaxially excited to avoid any performance-depreciating effects from the connectors.

**Fig. 2:**
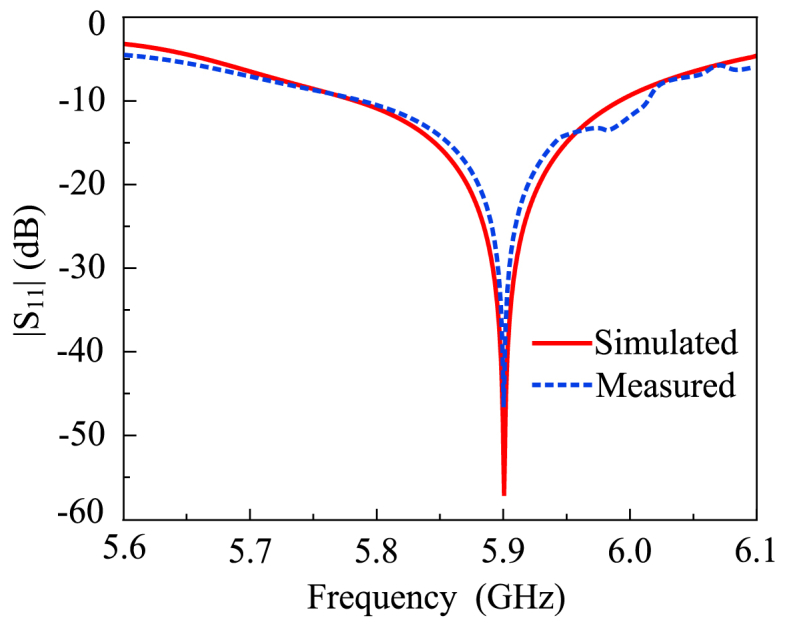
The |S_11_| (simulated and measured) characteristics of the proposed capacitively fed antenna.

Following that, a circular cleaving is established around the feeding position, and the diagonal corners of the radiating patch are truncated. The resulting effect broadens the bandwidth of the proposed antenna. The antenna is designed to resonate at the desired frequency of 5.9 GHz, fully covering the spectrum utilized for V2X communications in the world as presented in Fig. 2. The edge truncation in our design serves two primary purposes, it is employed to achieve precise operational frequency tuning and to prevent detrimental shorting with the parasitic element.

For a given transverse magnetic (TM) mode, the dimensions of the radiator can be initially calculated by analytical means based on the length and width of the patch [27]. The resonance frequency *f*_*mn*_ of a specific TM_*mn*_ mode can be evaluated as:

(1) $$f_{mn}=\frac{k_{mn}c}{2\pi \sqrt{\varepsilon_{r}}}$$

(2) $$k_{mn}=\sqrt{{\left(\frac{m\pi }{a}\right)}^{2}+{\left(\frac{n\pi }{b}\right)}^{2}}$$

Here, *c* refers to the speed of light in a vacuum, whereas *k*_*mn*_ refers to the wavenumber. In (2), *a* and *b* represent the radiator dimensions. There are other intrinsic factors of design as well like the effective dielectric constant. The effective dielectric constant also depends on the dielectric constant and the height of the substrate material used in the design. Table 1 presents a tabulation of the optimized parameters for the single-unit antenna. The selection of m and n determines the specific mode of operation and directly influences the antenna's resonant frequency. For a given mode, by adjusting the dimensions a and b, the wave number can be calculated, and consequently, the resonant frequency can be tuned to the desired value.

**Table 1:** Optimized parameters of the single-element antenna.

Parameter	Value (mm)	Parameter	Value (mm)	Parameter	Value (mm)
*P*	12.09	*M*	3	*L*_*s*_	36
*L*	12.09	*A*	6.52	*h*	1.52
*r*_*A*_	2.47	*r*_*B*_	2.3	*B*	1.4

**Fig. 3:**
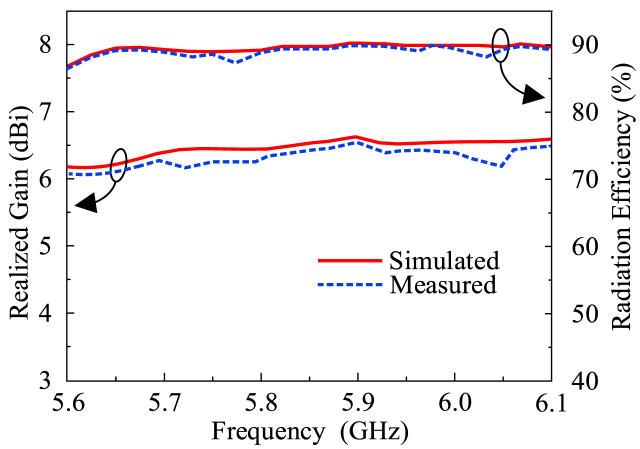
The simulated and realized gain and radiation efficiency of the single-element antenna.

**Fig. 4:**
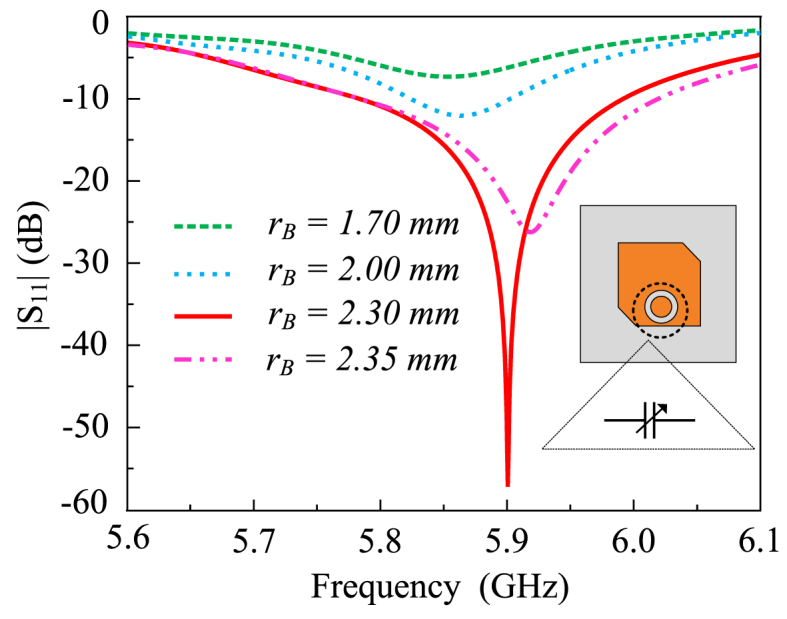
The antenna's |S_11_| characteristics with variation in *r*_*B*_.

The proposed design of the flat antenna with compactness, lightweight, and simple architecture allows for the production of prototypes on a large scale. The impedance matching of the antenna, corresponding to the |S_11_| < −10 dB is between 5.75 GHz and 6.0 GHz, fully covering the V2X spectrum recommended by the FCC and 5G Automotive Association (5GAA). The fractional bandwidth of 4.25 percent with respect to the central operating frequency is represented by the covered 250 MHz spectrum of the proposed antenna. The realized gain and radiation efficiency of the proposed antenna are also shown in Fig. 3. The single-unit antenna shows a high peak gain value of 6.48 dBi whereas the radiation efficiency is more than 90 percent across the operational region [28,29]. Fig. 4 presents the design and analytical modeling of antennas but the scope of the articles is to mathematically model the gap capacitance, substrate thickness-polarization tradeoff analysis, and retention of the polarization in radiating elements while maintaining a constant bandwidth of the antennas.

### 2.2 Parametric study of discoid gap capacitance

The annular slot on the radiating patch encompassing the feedline probe induces a capacitive effect. With an extensive parametric study of the gap, it is observed that the gap length is critical to the reflection coefficient amelioration at a specific frequency spectrum. The discoid gap capacitance *C*_*g*_ introduced by the slot can be calculated as (3) [29].

(3) $$C_{g}=2\pi r_{B}C_{l}$$

whereas the capacitance per unit length *C*_*l*_ can be calculated as (4)

(4) $$C_{l}\approx 2\frac{\varepsilon_{0}\varepsilon_{reff}}{\pi }$$

The effective permittivity $\varepsilon_{reff}$ is calculated as (5)

(5) $$\varepsilon_{reff}\approx 1+\frac{\varepsilon_{r}-1}{2}\;\frac{\ln\left(\frac{16h}{\pi \left(r_{A}-r_{B}\right)}\right)}{\ln\left(\frac{8r_{B}}{r_{A}-r_{B}}\right)}$$

**Fig. 5:**
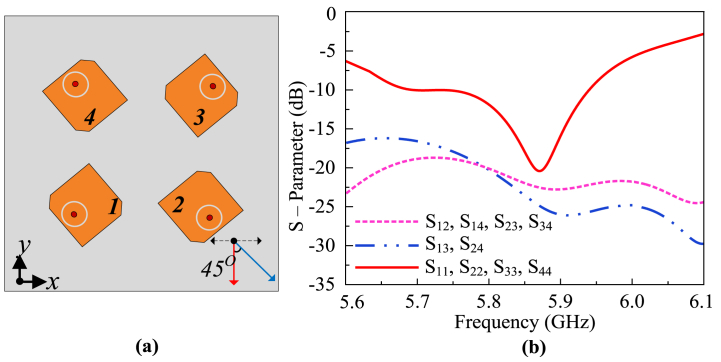
Configuration of the MIMO antenna (a) without hybrid decoupling mechanism and (b) respective S-parameters of the design.

At the value of $r_{A}-r_{B}=0.17$ mm, the highest impedance matching is observed. At the 0.17 mm slot gap, the capacitance value corresponds to approximately 0.8 pF. Fig. 5 shows the variations in the reflection coefficient of the proposed antenna with respect to changes in the $r_{B}$. As long as the slot persists and is in the primary radiating patch, the reflection coefficient improves to an extent with the increment of $r_{B}$. Above the 2.32 mm value of ${\;r}_{B}$, the reflection coefficient again starts to decrease, primarily due to a variation in the capacitance value. Furthermore, with the decrease in $r_{B}$ and an increment in the discoid gap capacitance *C*_*g*_, the reflection coefficient is depreciated and gradually results in a decrease in the impedance matching.

### 2.3 Design of the proposed MIMO antenna for V2X communications

The channel capacity of a system is substantially enhanced via MIMO technology. If the rate of information transmission is less than limit imposed by the channel capacity, information can be sent from transmitter to the receiver in a fading environment with minimal error rate. Thus, It is feasible to have an improved channel capacity for a communication system. MIMO technology greatly enhances the channel capacity, allowing reliability and spectral efficiency. The maximum channel capacity in a MIMO system is expressed in the form of equation (6) [30].

(6) $$C=\log_{2}\left(\det\left(I_{N}+\frac{\rho }{M}HH^{H}\right)\right)$$

**Fig. 6:**
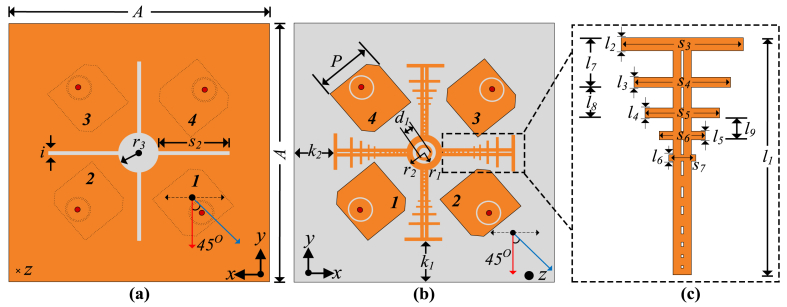
Geometry and configuration of the proposed MIMO antenna (a) defected ground plane (b) top view of patch and (c) magnified view of decoupling structure.

In above equation, C represents the capacity, ρ is signal-to-noise ratio, H is the channel matrix, whereas I represents the identity matrix. *M* and *N* represent the number of elements at transmitter and receiver, respectively. The channel capacity grows with the number of elements, making it useful for secure and fast communications. The proposed MIMO antenna design is a further development of the single element that was earlier described. The proposed MIMO antenna design configuration and geometry are depicted in Fig. 6. Initially, a 4-port MIMO antenna is designed without utilization of the hybrid decoupling mechanism. The antennas are positioned perpendicular to one another. Subsequently, in all lateral directions in space, the antennas are rotated 45° separate from one another. This allows beam coverage of more area. Fig. 5(a) represents the geometry of the antenna without the utilization of a hybrid decoupling structure, whereas Fig. 5(b) represents the respective S-parameters of the antenna. A parasitic decoupling hybrid structure is proposed to overcome mutual coupling and maintain the MIMO radiation performance of the antenna.

In a MIMO system, mutual coupling reduces the reliability of communication and adds errors to the transmission of information. For V2X communications, high data accuracy is a critical requirement. Therefore, mutual coupling should be reduced to ensure excellent data transmission accuracy [31]. Table 2 presents the optimized parameters of the proposed MIMO antenna for vehicular IoT systems.

**Table 2:** Optimized parameters of the proposed MIMO antenna.

Parameter	Value (mm)	Parameter	Value (mm)	Parameter	Value (mm)	Parameter	Value (mm)
*A*	56	*K*_*2*_	10	*l*_*1*_	15.45	*l*_*6*_	0.522
*i*	0.50	*K*_*1*_	10	*l*_*2*_	1	*l*_*7*_	3.55
*s*_*2*_	11.50	*r*_*2*_	3	*l*_*3*_	0.85	*l*_*8*_	2.167
*r*_*3*_	4	*r*_*1*_	2	*l*_*4*_	0.722	*l*_*9*_	1.84
*P*	12.09	*d*_*1*_	2.5	*l*_*5*_	0.614	*s*_*6*_	3.375
*s*_*3*_	8	*s*_*4*_	6	*s*_*5*_	4.50	*s*_*7*_	2.531

### 2.4 Design stages of quasi-fractal parasitic element and DGS

Mutual coupling distorts the radiation pattern of the antenna element. Furthermore, mutual coupling causes reduced isolation, increased side lobe levels, and impedance mismatching issues. As a result, a large number of studies have been developed to address this topic. Numerous methodologies include the utilization of parasitic elements [18,20,26], DGS [18,24], neutralization lines [32], and amalgamation of all these techniques.

A hybrid decoupling mechanism is utilized in the proposed design. By using a hybrid decoupling technique that combines DGS with a quasi-fractal parasitic element, the isolation between the individual elements is enhanced. Initially, a parasitic element is designed by perpendicularly orienting two stubs at the center of the antenna and a circular slot at the ground plane. The design is then modified to include T-shaped iterations in the parasitic element along with a central disc-shaped stub at the center. a linear slot is introduced at the ground plane in the same stage. The last stage involves designing two concentric circular slots at the center of the parasitic element and a plus-shaped slot at the ground plane. The decoupling design shows the highest isolation at the third step, which is the proposed design in this paper. Fig. 6(a) represents the defected ground structure, Fig. 6(b) represents the top view of the patch, whereas Fig. 6(c) presents the magnified view of the decoupling structure for the readers’ convenience.

**Fig. 7:**
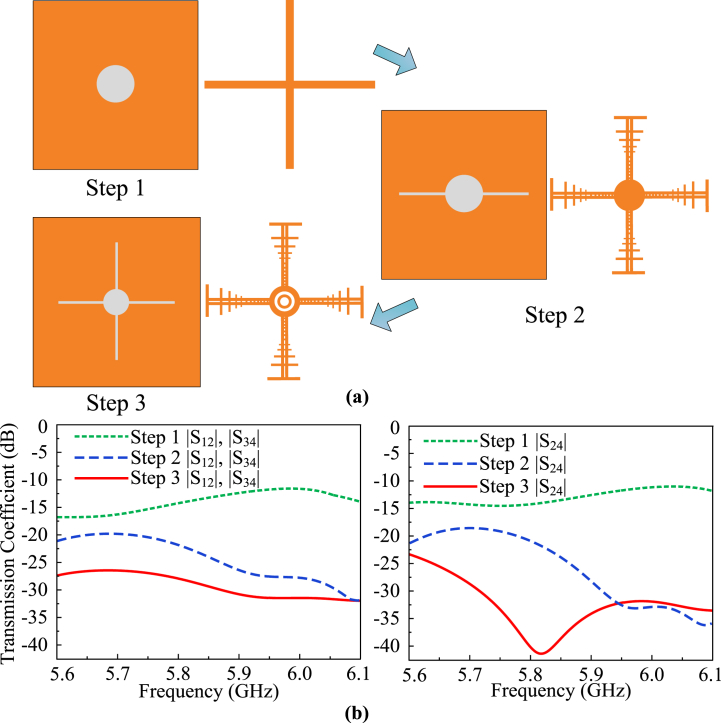
(a) Design stages of the decoupling mechanism and (b) respective S-parameters of the design.

The stages of the proposed geometry and configuration of the antenna, along with its respective S-parameters are shown in Fig. 7. As the proposed design is symmetrical along the vertical and horizontal axis, the transmission coefficients (|S_13_|, |S_24_|, |S_31_|, |S_42_|) of the diagonal radiating elements are approximately alike. Additionally, the transmission coefficients of the vertically oriented radiating elements (|S_12_|, |S_21_|, |S_14_|, |S_41_|, |S_23_|, |S_32_|, |S_43_|, and |S_34_|) are fairly comparable. Thus, only (|S_13_|,|S_34_| and |S_24_|) are shown in Fig. 7(b) for the sake of simplicity for the reader.

To understand the functionality of the decoupling structure, surface current distributions are studied at 5.9 GHz frequency. Fig. 9 shows the surface current distribution at 5.9 GHz when one of the MIMO antenna units is electrically excited. Mutual coupling arises when current is inducted from one element to another. This lowers the performance characteristics of the MIMO antenna. The decoupling structure is used to minimize current flow between elements, which lowers current couplings and improves the isolation. The hybrid decoupling technique combining the DGS and the quasi-fractal parasitic element leverages the strengths of both methods to achieve superior mutual coupling suppression. The DGS provides a localized stopband effect, while the parasitic element introduces reactive fields that counteract coupling.

### 2.5 MIMO antenna performance comparison with and without proposed decoupling structure

**Fig. 8:**
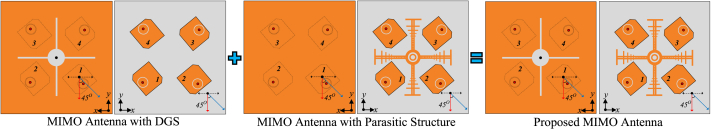
Design perspective of the proposed MIMO antenna using a DGS and a parasitic element.

The results of the MIMO antenna, mainly the transmission coefficients, are shown in Fig. 8. As evident by the transmission coefficients, it is noted that the mutual coupling is reduced, thereby improving the isolation. This design approach is akin to metamaterials, which are typically structured to achieve specific electromagnetic responses not found in natural materials. The parasitic element alters the effective electromagnetic response in a manner comparable to metamaterials by introducing additional reactive fields and modifying the coupling behavior between antenna elements. Surface current distribution analysis and transmission coefficient offers an insight into the electromagnetic interactions within the antenna structure. By examining how currents are distributed and how they interact with the parasitic element, we can validate the effectiveness of the design in minimizing unwanted couplings and optimizing interelement interaction. Furthermore, extensive parametric analysis is performed to optimize the behavior of the antenna module. The S-parameters of the proposed antenna is presented in Fig. 10. As the proposed design is symmetrical along the vertical and horizontal axis, the transmission coefficients (|S_13_|, |S_24_|, |S_31_|, |S_42_|) of the diagonal radiating elements are approximately alike. Additionally, the transmission coefficients of the vertically oriented radiating elements (|S_12_|, |S_21_|, |S_14_|, |S_41_|, |S_23_|, |S_32_|, |S_43_|, and |S_34_|) are also approximately equal. Thus, only (|S_11_|, |S_13_| and |S_24_|) are shown in Fig. 8 for the sake of simplicity for the reader.

**Fig. 9:**
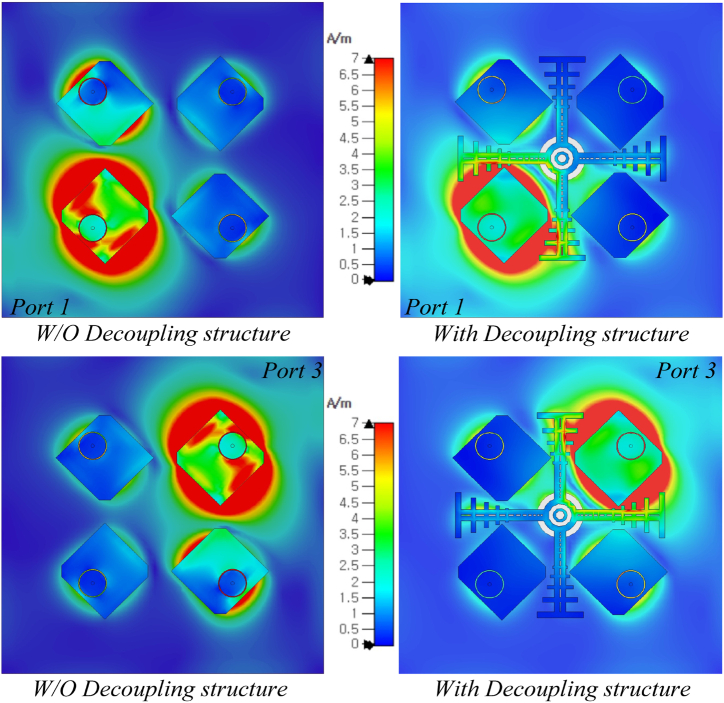
The surface current of different ports at 5.9 GHz frequency.

**Fig. 10:**
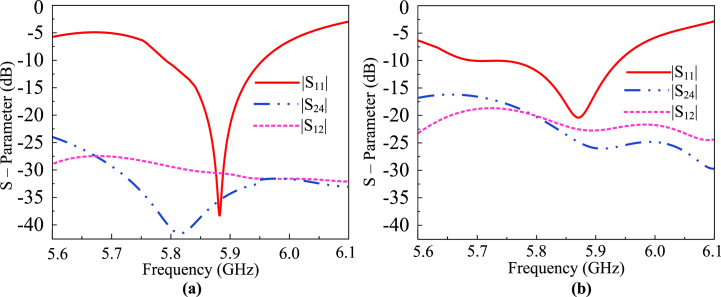
The S–parameters of the proposed V2X MIMO antenna (a) with and (b) without decoupling structure.

## 3 Measured results and discussion

**Fig. 11:**
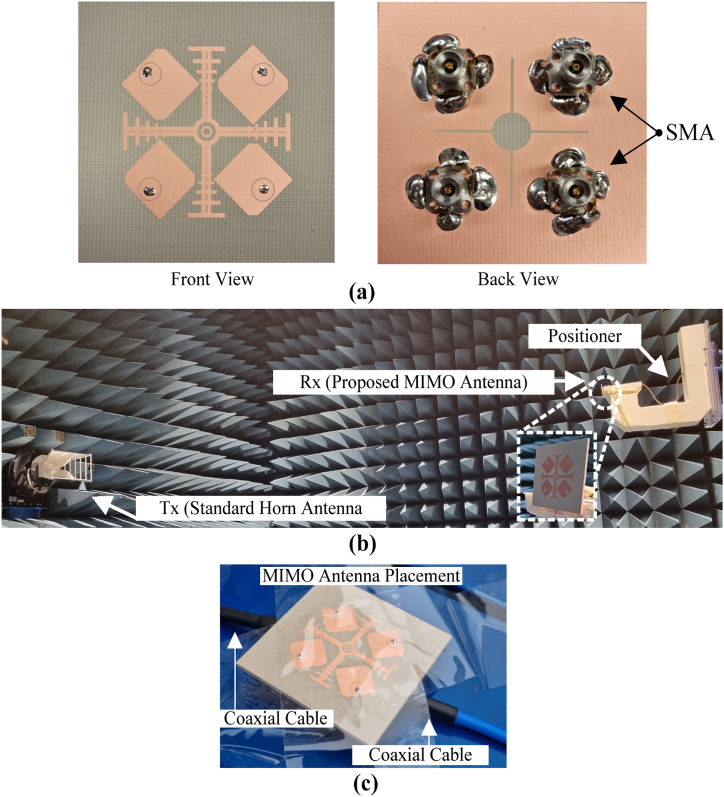
Photographs of the (a) Fabricated prototype of the proposed V2X antenna (b) Far-field measurements setup and (a) Placement of antenna on passenger car.

**Fig. 12:**
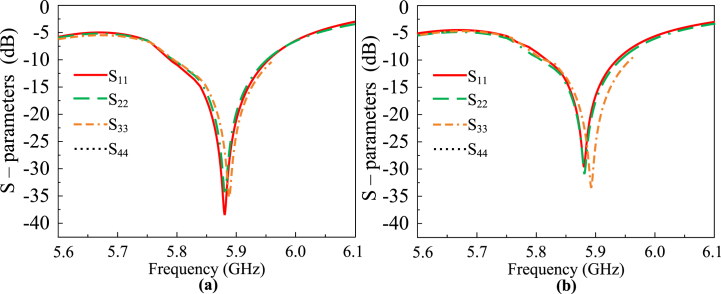
The S – parameters of the proposed V2X MIMO antenna (a) simulated and (b) measured by placement on the passenger car.

For the validation of the design, a physical prototype is designed on the substrate Rogers RO4350B ($\varepsilon_{r}$ = 3.66 and tanδ = 0.0037) with a height of 59.85 mils. A 50-Ω coaxial connector is used to feed the radiating patch. To measure the S-parameters, a network analyzer, Agilent Technologies E8364B is used, placed inside a car. Furthermore, an electromagnetically isolated anechoic chamber is employed to validate the far-field properties. A well-calibrated high-gain horn antenna is utilized as a transmitter for the far-field tests. The measurement setup is shown in Fig. 11(b). The antenna is rotated 360° to find the realized gain at all the points in space. The antenna shows an impedance bandwidth of 200 MHz, ranging from 5.77 GHz to 5.97 GHz. During the tests, only a single port of the MIMO antenna is excited, while the other ports are terminated with a 50-Ω load. The peak realized gain of the MIMO antenna is 6.5 dBi, which is above the peak gain of the single-element antenna. When all the antennas are excited simultaneously, a peak realized gain value of 3.85 dBi is observed. The individual and collective excitation allows 180 degrees of pattern diversity. Fig. 12 depicts the measured and simulated S-parameters (|S_11_|, |S_22_|,|S_33_| and |S_44_|). There is an agreement between simulated and measured results. Therefore, the design is a considerable candidate for enabling IoT in automotive systems by Vehicle-to-Device (V2D), Vehicle-to-Grid (V2G), Vehicle-to-Infrastructure (V2I), Vehicle-to-Vehicle (V2V), and Vehicle-to-Pedestrian (V2P) communications paradigms.

### 3.1 Reflection coefficients

Since each antenna is oriented symmetrically with the other ones, their reflection coefficient curves are nearly identical. The bandwidth of the proposed MIMO antenna for V2X applications is also approximately equal to that of a single element, completely encompassing the V2X spectrum that the FCC and 5G Automotive Association (5GAA) have recommended.

The simulated and measured S-parameters of the proposed antenna are shown in Fig. 12. For S–parameters measurements, a practical car body (aluminum sheet beneath the antenna surface) is used. The MIMO antenna is placed at the rear side of the car roof, fed by a 250 mm coaxial cable connected to the Vector Network Analyzer for measurements.

### 3.2 Transmission coefficients

**Fig. 13:**
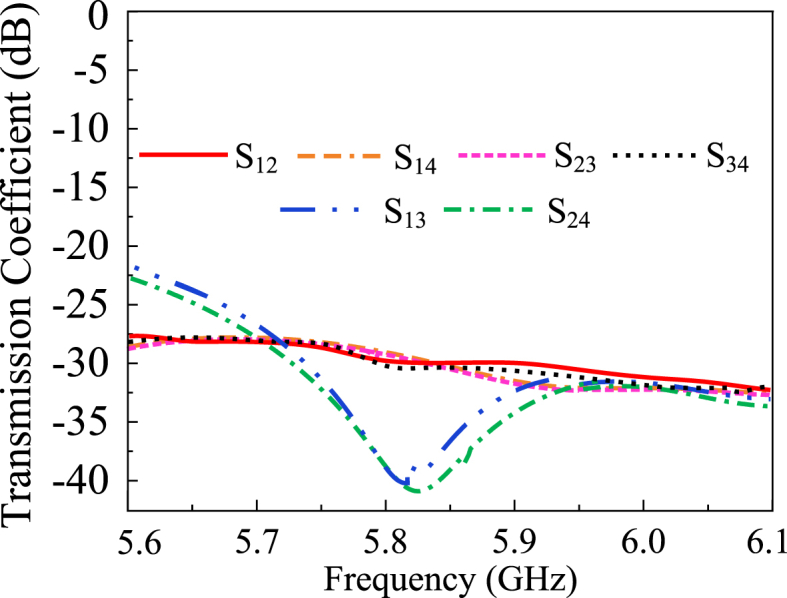
The transmission coefficient of the proposed V2X MIMO antenna.

The transmission coefficients of the proposed MIMO antenna with a decoupling structure are depicted in Fig. 13. As per the analysis, the suggested antenna offers high isolation characteristics. The measured minimum isolation offered by the proposed design is 28 dB, with a maximum value of 43 dB over the operating frequency.

### 3.3 Radiation pattern and beam coverage

**Fig. 14:**
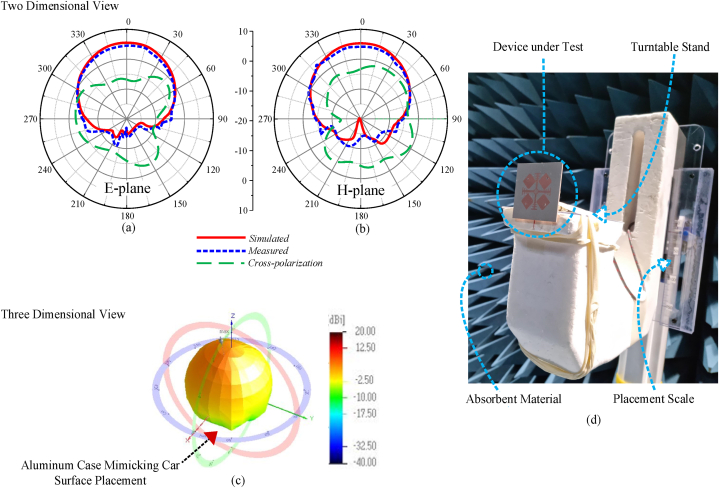
Radiation pattern of the proposed MIMO antenna (a) 5.9 GHz at E-plane and (b) 5.9 GHz at H-plane (c) Measured 3D radiation pattern with a case mimicking car surface and (d) positioner within an anechoic chamber.

The proposed MIMO antenna exhibits a directional radiation pattern. With a directional radiation pattern, the energy is projected in a specific direction, thus having more energy concentration on a specific subspace. The proposed MIMO antenna delivers low levels of back lobes, and it is capable of integration with the car roof. Fig. 14 (a) and (b) show the two-dimensional radiation pattern, showing measured and simulated radiation pattern. To assess the three-dimensional radiation propagation in space. Fig. 14 (c) shows the measured radiation pattern in space. An aluminum case of dimensions 250 mm × 250 mm is first placed prior to the antenna surface to introduce a surface effect of vehicle roof on the antenna in an anechoic chamber to mimic the car roof body in a practical scenario. Table 3 tabulates the measured values of realized gain at specific port. It is significant to note that when all of the ports are stimulated at once, the subspaces where the beam is not propagating while a particular port is excited are encompassed.

**Table 3:** Compared gains of the proposed V2X MIMO antenna.

Simulated values					Measured values				
Port 1	Port 2	Port 3	Port 4	Simultaneous excitation	Port 1	Port 2	Port 3	Port 4	Simultaneous excitation
6.50	6.51	6.49	6.51	3.97	6.48	6.5	6.43	6.47	3.87

In our experimental setup, the 3D radiation pattern measurements were conducted in an electromagnetically isolated anechoic chamber to ensure accurate and reliable results. The antenna was precisely positioned on a turntable that allowed for full 360-degree rotation, enabling the capture of radiation patterns in all directions. A calibrated reference antenna was used to validate the measurements, and the entire setup was electromagnetically isolated to minimize interference and reflections. The measurement process involved recording the radiation patterns at multiple frequencies across the operational bandwidth to assess the antenna's performance comprehensively. The beam direction in a given area may be dynamically controlled by an embedded computer using an SP4T RF switch module that is connected to a Universal Serial Bus (USB) module. Fig. 14 (d) shows the designed prototype in an anechoic chamber rotating by a turntable. The proposed antenna has low cross-polarization indicated in 3D radiation pattern. Reduction of polarization can help reduce interference between adjacent antennas. As reported in Refs. [33,34], cross-polarization suppression can be achieved with using slot feeds and specialized graphene-based inks. With inclusion of such specific techniques into the design, more cross-polarization can be observed.

**Fig. 15:**
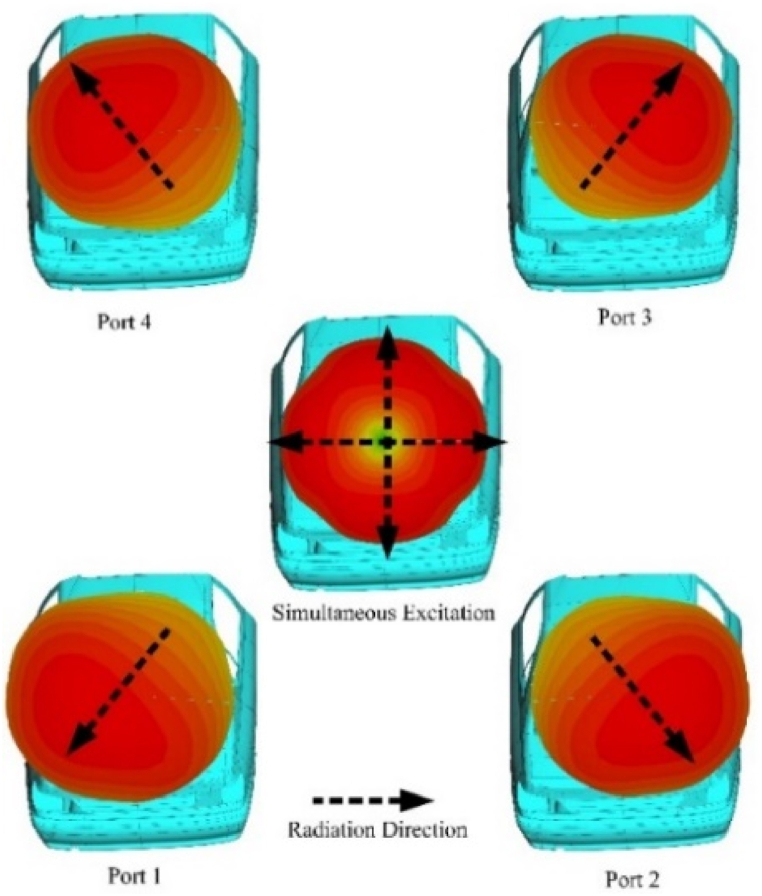
Antenna radiation pattern on a car model for specific port excitation and radiation diversity.

Fig. 15 shows the relative propagation direction when each port is excited. With each port excited independently, the direction of propagation can be controlled in a specific direction .Whereas for propagation of electromagnetic waves in directions which are not achievable through individual ports, all ports are excited simultaneously to effectively cover the lacking areas in space for the propagation that cannot be achieved by individual ports.

### 3.4 Envelope correlation coefficient

**Fig. 16:**
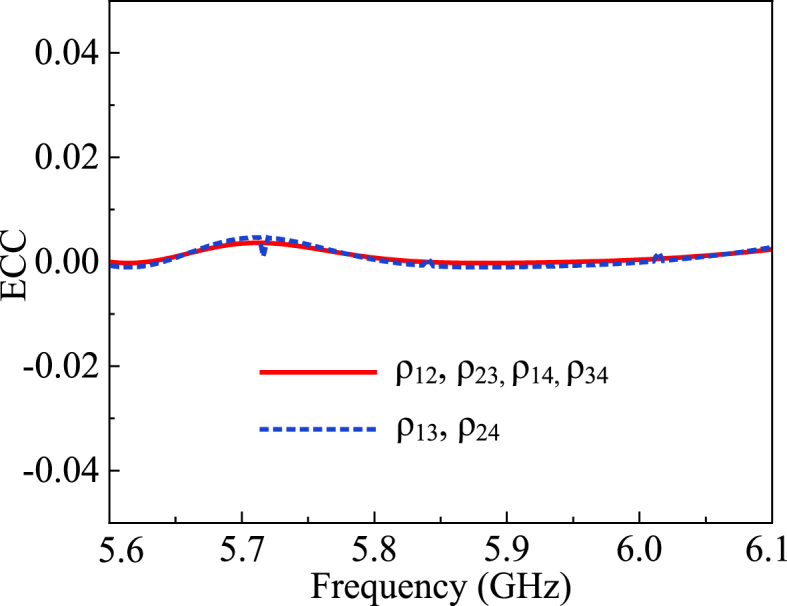
The envelope correlation coefficient of the proposed MIMO module.

The envelope correlation coefficient (ECC) is the measure of correlation between the radiation patterns of two antenna elements. It is an important performance metric for MIMO systems, especially for spatial multiplexing. ECC is inversely proportional to the diversity gain. The higher value of ECC implies that the antennas are highly correlated. To maximize spatial multiplexing, a low ECC value is desirable. The ideal value of ECC is zero, whereas the maximum tolerable value of ECC is 0.5 for physical scenarios. The proposed MIMO antenna for the V2X application has a calculated ECC value of below 0.001 for the operational frequency as shown in Fig. 16. Equations (7), (8) [35] provide the value of ECC in terms of transmission coefficient, and far-field radiation patterns, respectively.

(7) $$\rho_{eij}=\frac{{\left|S_{ii}*S_{ij}+S_{ji}*S_{jj}\right|}^{2}}{\left(1-{\left|S_{ii}\right|}^{2}-{S_{ij}}^{2}\right)\left(1-{\left|S_{ji}\right|}^{2}-{S_{jj}}^{2}\right)}$$

(8) $$\rho_{eij}=\frac{{\left|\iint_{0}^{4\pi }\left[\vec{R_{i}}\left(\theta ,\varphi \right)\times \vec{R_{j}}\left(\theta ,\varphi \right)\;\right]d\Omega \right|}^{2}}{\iint_{0}^{4\pi }{\left|\vec{R_{i}}\left(\theta ,\varphi \right)\right|}^{2}d\Omega \iint_{0}^{4\pi }{\left|\vec{R_{j}}\left(\theta ,\varphi \right)\right|}^{2}d\Omega \;}$$

### 3.5 Diversity gain

**Fig. 17:**
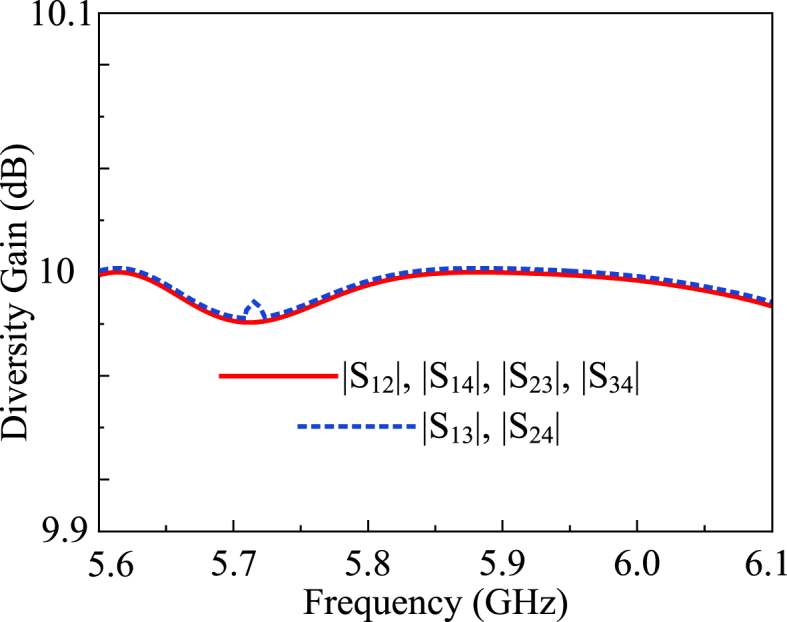
The diversity gains of the proposed MIMO antenna.

Diversity Gain (DG) refers to the improvement in the signal quality by the utilization of multiple antennas. When the transmitter sends transmission signals to the receiver antennas, it experiences fading conditions. To mitigate the effect of fading and reduce the bit error rate (BER), the signals received at different antennas are combined. This improves the overall signal reception characteristics. It can be observed in Fig. 17 that, the proposed MIMO antenna exhibits an approximately ideal DG value of 10 dB. Mathematically, DG is expressed in the form of equation (9) [36].

(9) $$\text{DG}=10\sqrt{{1-\left|\rho ij\right|}^{2}}$$

### 3.6 Mean effective gain (MEG)

**Fig. 18:**
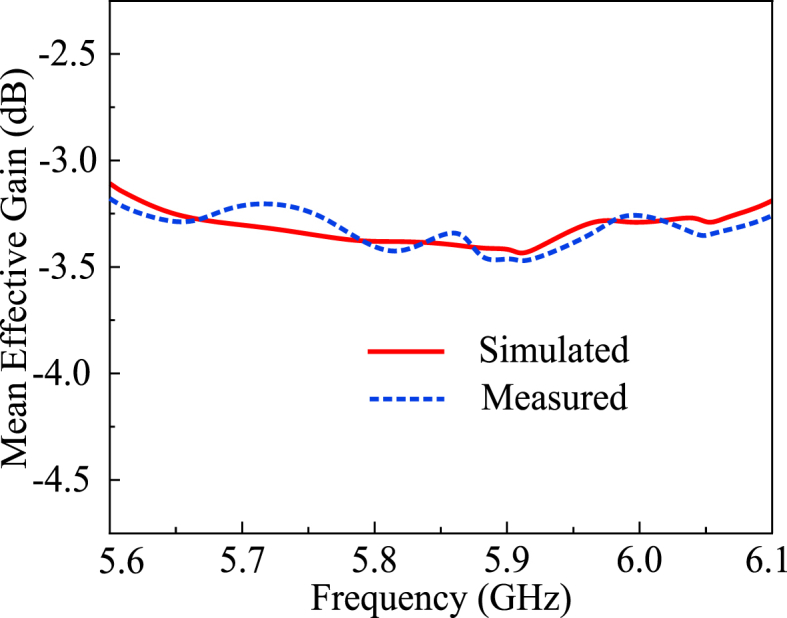
The mean effective gain of the proposed MIMO antenna.

For a MIMO antenna to perform, the applicable value of MEG should be below −3 dB. As per Fig. 18, the MEG value is below the threshold of −3 dB throughout the operational frequency. For *N* number of antennas in a MIMO system, the MEG can be calculated by the summation expression in (10) [36].

(10) $${MEG}_{i=0.5}\left(1-\sum_{i=1}^{N}\left|S_{ij}\right|\right)$$

### 3.7 Channel capacity loss (CCL)

**Fig. 19:**
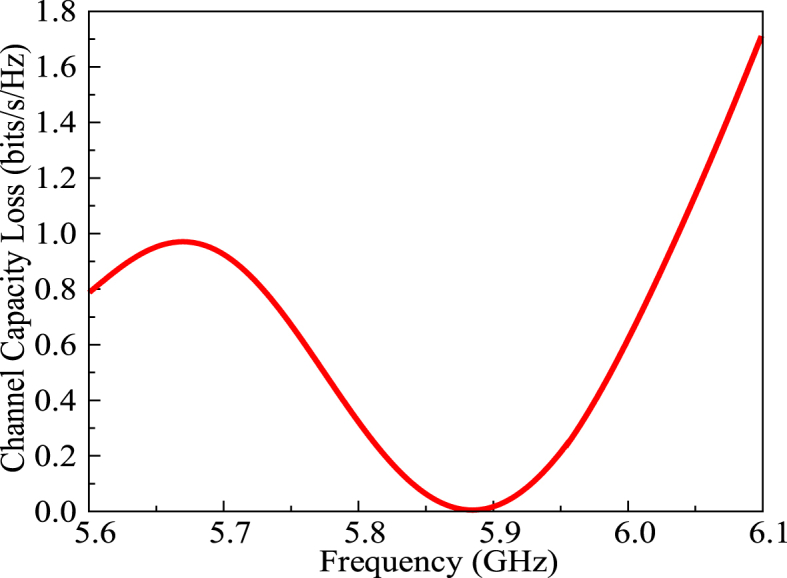
The CCL of the proposed MIMO antenna.

The channel capacity loss of the proposed MIMO antenna is depicted in Fig. 19. For operational characteristics, a MIMO antenna must have CCL below 0.4 bits/sec/Hz.

### 3.8 Total active reflection coefficient (TARC)

**Fig. 20:**
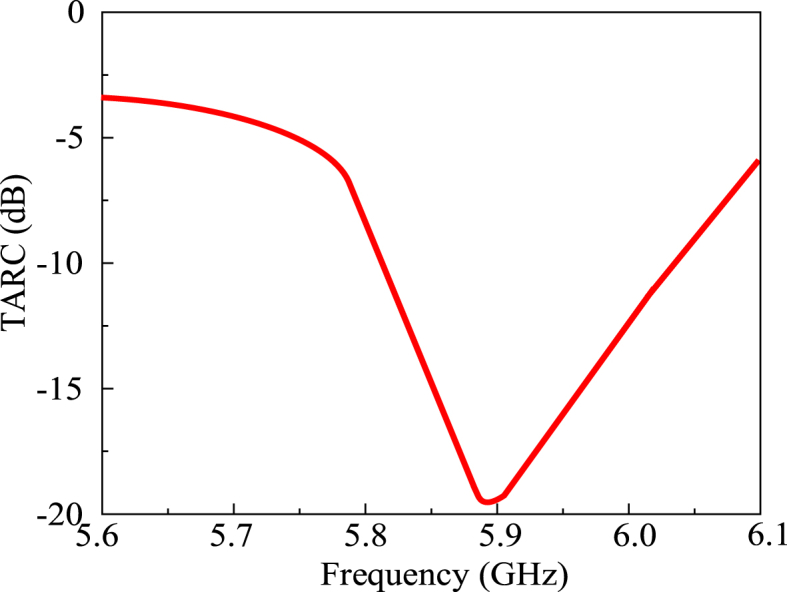
The TARC of the proposed MIMO antenna.

The TARC of the proposed MIMO antenna is depicted in Fig. 20.

### 3.9 Link budget analysis and transmitter power limits

V2X technologies in vehicular communications are subject to regulatory maximum power transmission constraints. Table 4 tabulates the characteristics of the radiated fields for technologies utilizing V2X communications paradigms. V2X would remain an essential technology for autonomous vehicles [37]. V2X is particularly important in road works warnings, turn-assist, pedestrian and other road users’ identification systems, and blind curve detections.

**Table 4:** V2X communications at a glance.

Technology	Application	Frequency Band (GHz)	Channel Bandwidth	Maximum Transmit Power (dBm)
LTE-V2X by PC5/Uu Interface	Non-safety and safety information communication	5.855–5.925	10/20 MHz	23 (measured in 1 ms)
IEEE 802.11p/ITS-G5	Non-safety and safety information communication	5.855–5.925	10 MHz	33 in EU and US for non- government services 44.8 in US for government services
Dedicated Short-Range Communications (DSRC)	Transport and telematics systems	5.795–5.815	0.5 MHz	14 or 21 (depending upon on- board unit)
5G-V2X	Non-safety and safety information communication	5.855–5.925/3.5	20 MHz	23

In a communication system, link budget analysis takes into consideration the gains and losses that the signal experiences through a communication medium such as waveguides, optical fibers, or coaxial cables. To determine the appropriateness of the proposed MIMO antenna for V2X scenarios, the link budget is calculated by the equation (11) [38].

(11) $$P_{RX}=P_{TX}+G_{TX}-L_{TX}-L_{FSP}+L_{M}+G_{RX}-L_{RX}$$

Here, $P_{RX}$ and $P_{TX}$ refer to the received and transmit powers refers, $L_{TX}$ refer to the system degradation losses due to mismatch and component losses at the transmitter end, $L_{M}$ account for the multifarious losses like polarization misalignment, fade margin, etc. $L_{RX}$ are the system degradation losses at the receiver end. $G_{RX}$ and $G_{TX}$ are the antenna gains at the receiver and transmitter sides, respectively. To assess the link budget, only $L_{FSP}$ is the variable parameter with respect to the propagation distance and central frequency of the antenna. $L_{FSP}$ can be assessed by equation (12) [39].

(12) $$L_{FSP\left(\text{dB}/\text{Km}\right)}=32.4+20\;\log_{10}\left(d_{\text{Km}}\right)+20\;\log_{10}\left(f_{\text{MHz}}\right)$$

A python script is used to calculate the link budget using *matplotlib* library for plotting the data by using the aforementioned equations.

The minimum detectable signal with can be calculated by the equation (13).

(13) $$DS=10\;\log_{10}\left(\frac{kT}{1mW}\right)+NoiseFigure+10\;\log_{10}\left(BW\right)$$

**Fig. 21:**
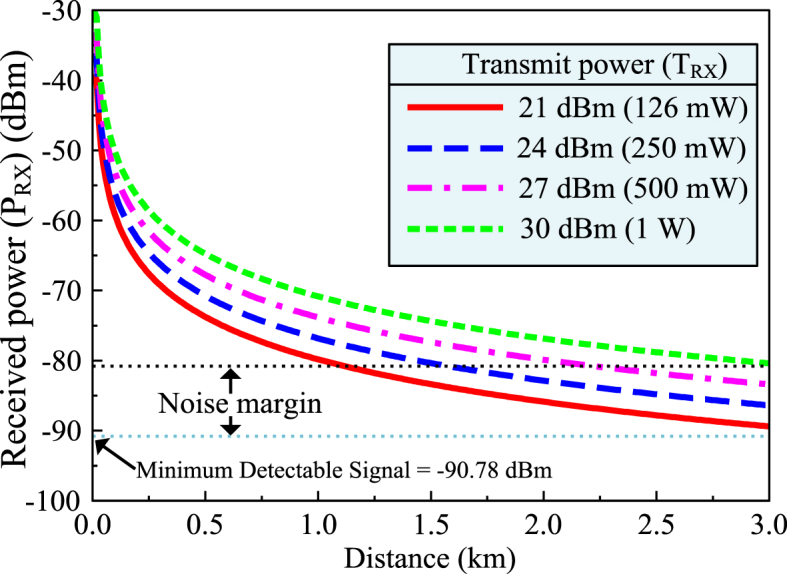
Link budget of the proposed MIMO antenna for different transmit power.

In the above equation, DS refers to the minimum value of detectable signal. BW is the bandwidth of operation, and *k* is the Boltzmann constant. *T* is the temperature of the environment where the antenna is placed (290 K). Using a noise margin of 10 dB, the proposed MIMO antenna offers a minimum sensitivity of −80.78 dBm. The World Health Organization has limited the transmission power of an antenna to 1 W in urban settings. With a minimum power of 126 mW, the antenna allows a signal transmission distance of 1.3 Km. The signal transmission distance conforms to the requirements set by FCC for maximum transmitter power. In a similar vein, the transmission distance of the communication signals may be extended up to 3 Km at a maximum strength of 30 dBm (1 W). Fig. 21 shows the link budget of the suggested MIMO antenna for V2X communications.

The human body is composed of dispersive tissues. The primary contributory factor to this is the variations in the material characteristics of the body's cells. Equation (14) is an empirically derived equality of the dielectric properties of the bodily tissues with respect to frequency [40,41].

(14) $$\epsilon \left(\omega \right)=\epsilon_{\infty }+\sum_{n}\frac{\Delta \epsilon_{n}}{1+{\left(j\omega \tau_{n}\right)}^{1-\alpha_{n}}\;}+\frac{\sigma_{i}}{j\omega \epsilon_{0}}$$

**Fig. 22:**
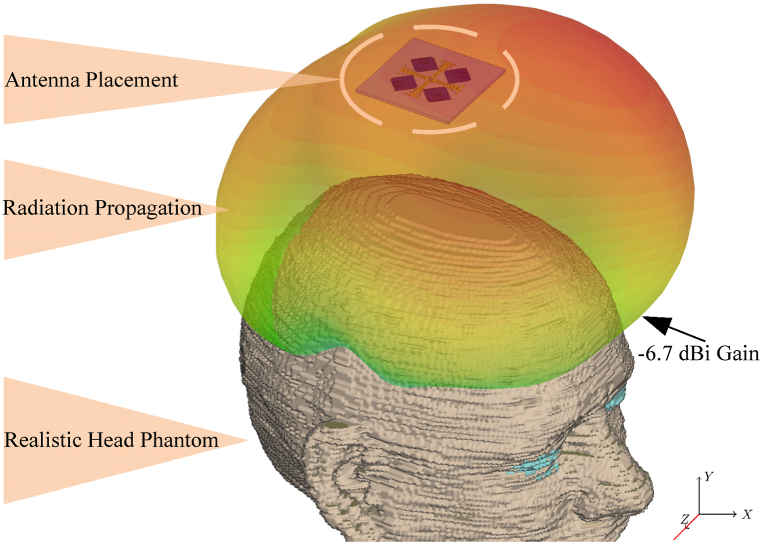
Radiation propagation on realistic head model.

Fig. 22 shows antenna placement on a realistic head model, which is based on equation (14). The human head voxel is derived from Ref. [42], and is based on high-resolution MRI images. To achieve precision in simulation setups, dispersive properties of head tissues are utilized in the design. The Transmission Line Matrix (TLM) is used is CST software, which is a time domain solver to assess 3D EMC applications. For simulation settings, the worst-case scenario—which includes no roof material in the event that the car's roof is open—has been employed. −6.87 db simulated gain is found at the driver head when the antenna is positioned 80 mm above the head voxel. Thus, low back lobe radiation in the proposed design is appropriate for long-term radiation exposures with minimal impact on human body.

## 4 Performance comparison with previous work

A performance comparison is tabulated in Table 5 between the proposed MIMO antenna for V2X applications and the existing literature of antenna design. In Ref. [16], a three-element MIMO antenna is presented with self-decoupled probing for V2X communications. The minimum isolation offered by the design is more than 24 dB. However, the spacing between the antenna elements is not compact. In Ref. [17], field decorrelations are used in a MIMO antenna to offer an isolation of more than 16 dB. However, the isolation achieved by the design may not be suitable for V2X communications, which require high values of isolation [18]. utilizes a parasitic element to achieve high isolation at the cost of more interelement spacing [19,22]. offer self-decoupled patches for isolation enhancement. However, the spacing imposed by the design may restrict the use cases of the proposed MIMO antennas.

**Table 5:** The proposed MIMO antenna system and published related works: A comparison.

Ref.	Isolation Enhancement Technique	Central Frequency (GHz)	Bandwidth (GHz)	Number of Ports	Minimum Inter-element Spacing	Minimum Isolation (dB)	ECC	Radiation Efficiency (%)	Peak Gain (dBi)	Gain at Head Voxel (Worst Case)
[16]	Self-Decoupled Probing	2.5	2.2–2.8	3	0.17 λ	>24	<0.005	90		–
[17]	Field Decorrelation by PRS	5.37	5.27–5.46	2	0.14 λ	>16	–	–	9.12	–
[18]	DGS + Patch	5.9	5.61–6.02	4	0.17 λ	>34	<0.001	94	7.68	–
[19]	Self-Decoupled Patch	5.9	5.85–5.95	2	≈0.36 λ	>29	<0.001	95	8.3	–
[20]	Parasitic Element	5.4	5.0–5.8	2	0.09 λ	>20	<0.02	95	6.8	–
[21]	Gap-Coupled Shortening Strips	4.15	3.3–5.0	4	0.554 λ	>13	<0.05	84	6.8	–
[22]	Self-Decoupled Patch	3.5	1.5–5.5	4	0.07 λ	>15	<0.016	84	3	–
[23]	L-Shaped Metal Walls Integrated	4.15	3.3–5.0	4	0.301 λ	>15	<0.03	80	7.5	–
[24]	DGS + Reflector	5.9	5.84–6.01	4	≈0.2 λ	>16	<0.028	75	6.3	–
[25]	Structurally Independent Orientation	5.9	0.61–6.0	4	0.25 λ	>26	<0.39	70	4.9	–
[26]	Microstrip Resonator	5.9	5.84–6.08	2	0.46 λ	>35	–	–	4.98	–
[43]	PIFA with Decoupling Network	5.9	5.3–6.75	4	≈0.20 λ	>10	–	–	–	–
[44]	Parasitic Element with Patch	5.8	5.53–7.32	4	≈35 λ	>29	<0.001	98	4.63	–
This	DGS + Parasitic Element	5.9	5.77–6.0	4	0.078 λ	>28	<0.001	90	6.5	−6.87 dB

Moreover, in Ref. [21], gap-coupled shortening strips are used to generate decoupling waves for isolation enhancement. The design offers a minimum isolation of more than 13 dB. This technique can be applied to applications where decoupling wave generation can play a vital role in mutual coupling suppression. However, the proposed design offers less isolation which may not be suitable for high-performance V2X scenarios.

Three-dimensional structures outside the main antenna geometry have been incorporated in MIMO antennas for mutual coupling suppression. In Ref. [23], an L-shaped metal wall is integrated with the main design geometry to enhance the isolation, and a reflector is incorporated at the rear side of the antenna to suppress mutual coupling in Ref. [24]. With improved complexity in design geometry, this integration can improve the isolation between individual elements. Furthermore, in Ref. [26], a microstrip resonator is etched between the antenna elements to enhance the surface current distribution and ameliorate the isolation between individual antenna elements. With a high value of interelement spacing and practically less value of isolation, the proposed designs may lack in terms of performance requirements in V2X applications.

[25] proposes an automotive MIMO antenna using a low profile PIFA with an interelement spacing of 0.25 λ. The antenna uses a stark fin housing to incorporate the antenna on the car structure. However, the interelement spacing imposed by the antenna and a relatively less value of gain, radiation efficiency and isolation may limit the practicality of the proposed design in Ref. [25] for V2X applications.

In summary, the proposed MIMO antenna for vehicular IoT in this paper with its geometrically straightforward architecture, high isolation, excellent radiation efficiency, 180° beam coverage, low back lobes, high realized gain and a minute value of ECC renders it as a valuable design for future V2X applications.

## 5 Conclusion

In this paper, a MIMO antenna module is proposed for vehicular IoT communications. Using a noise margin of 10 dB, the proposed MIMO antenna offers a minimum sensitivity of −80.78 dBm. The measured minimum isolation offered by the proposed design is 28 dB, with a maximum value of 43 dB over the operating frequency. The proposed antenna offers geometrically straightforward design architecture, utilization of defected ground structure (DGS), and quasi-T shaped parasitic element for reduction of electromagnetic interactions between the antenna elements. This simultaneous use of DGS and parasitic elements results in mutual coupling suppression, a crucial requirement for safety-critical communication systems. The individual antennas are oriented to achieve 180 degrees of beam coverage. The suggested antenna offers minimal ECC value, strong isolation, outstanding radiation efficiency, 180-degree beam coverage, low back lobes, high realized gain, and geometrically simple construction making it a useful design for upcoming V2X applications.

## CRediT authorship contribution statement

**Syed Naheel Raza Rizvi:** Writing – original draft, Formal analysis, Data curation, Conceptualization. **Md Abu Sufian:** Validation. **Niamat Hussain:** Supervision, Software. **Yangbae Chun:** Visualization. **Seong-Gyoon Park:** Project administration, Investigation. **Sunggoo Kim:** Supervision. **Nam Kim:** Project administration, Methodology, Formal analysis.

## Data availability statement

**Table:** 

Data and Code Availability Has data associated with your study been deposited into a publicly available repository?	No
Please select why. Please note that this statement will be available alongside your article upon publication. Has data associated with your study been deposited into a publicly available repository?	**Data included in article/supp. material/referenced in article**

## Declaration of competing interest

We do not have any conflicts of interest associated with this publication, and there has been no significant financial support for this work that could have influenced its outcome. As Corresponding Author, I confirm that the manuscript has been read and approved for submission by all the named authors.
